# Conversations between Drs. Warren and Putnam on the Subject of Medical Ethics

**Published:** 1884-08

**Authors:** 


**Conversations Between Drs. Warren and Putnam on the Subject of Medical** Ethics. With an Account of the Medical Empiricisms of Europe and America. By FRANK H. HAMILTON, M. D. Bermingham & Co., 28 Union Square, New York. 1884.

Anything from the pen of Dr. Hamilton is worth reading, and in this little book he has condensed and attempted to fairly represent the prevailing opinions of medical men in this country on the subject of the code. Knowing the author's positive opinion upon the subject, it is not surprising that he makes Dr. Putnam, the representative of the old code, get the best of the argument.

